# Experimental Study of the Effect of Corneal Bio-Coating Based on the Original Hydrogel Biopolymer Scaffold on the Anterior Segment Structures of the Eye

**DOI:** 10.17691/stm2025.17.4.04

**Published:** 2025-08-29

**Authors:** I.N. Grigoryeva, N.A. Akmalov, A.V. Shatskikh, A.A. Voskresenskaya, M.N. Egorikhina, Yu.P. Rubtsova, N.A. Pozdeyeva

**Affiliations:** Ophthalmologist, Department of Reconstructive Oculoplastic and Laser Surgery; S. Fyodorov Eye Microsurgery Federal State Institution, Cheboksary Branch, 10 Traktorostroiteley Avenue, Cheboksary, 428028, Russia; Resident Physician; Postgraduate Doctors’ Training Institute in the Chuvash Republic, 27 Mikhail Sespel St., Cheboksary, 428018, Russia; MD, PhD, Head of the Laboratory of Pathologic Anatomy and Histology of the Eye; S. Fyodorov Eye Microsurgery Federal State Institution, Beskudnikovsky Boulevard, Moscow, 127486, Russia; MD, PhD, Deputy Director for Scientific Work; S. Fyodorov Eye Microsurgery Federal State Institution, Cheboksary Branch, 10 Traktorostroiteley Avenue, Cheboksary, 428028, Russia; Associate Professor, Department of Surgery with the Course of Ophthalmology; Postgraduate Doctors’ Training Institute in the Chuvash Republic, 27 Mikhail Sespel St., Cheboksary, 428018, Russia; PhD, Head of the Scientific Laboratory of Cell Technologies, Research Institute of Experimental Oncology and Biomedical Technologies; Privolzhsky Research Medical University, 10/1 Minin and Pozharsky Square, Nizhny Novgorod, 603005, Russia; PhD, Researcher, Scientific Laboratory, Research Institute of Cell Technologies, Research Institute of Experimental Oncology and Biomedical Technologies; Privolzhsky Research Medical University, 10/1 Minin and Pozharsky Square, Nizhny Novgorod, 603005, Russia; MD, DSc, Professor, Director; S. Fyodorov Eye Microsurgery Federal State Institution, Cheboksary Branch, 10 Traktorostroiteley Avenue, Cheboksary, 428028, Russia; Professor, Department of Surgery with the Course of Ophthalmology; Postgraduate Doctors’ Training Institute in the Chuvash Republic, 27 Mikhail Sespel St., Cheboksary, 428018, Russia

**Keywords:** cornea regeneration, hydrogel scaffold, scaffold

## Abstract

In recent years, there have been proposed various kinds of bioengineering constructions of scaffolds — biomaterial structures serving as substrates for restoration and regeneration of tissues. They possess marked biocompatibility, are capable of supporting high concentration of substances delivered to the injured tissues, and may be standardized in the process of fabrication.

**The aim of the investigation** is to study experimentally the effect of the bio-coating based on the original hydrogel biopolymer scaffold on the intact structures of the anterior segment of the eye and its adnexa and evaluate its safety.

**Materials and Methods:**

The experimental study was carried out on 6 rabbits (6 eyes). The original hydrogel biopolymer scaffold of 10.0 mm in diameter, 1.5 and 2.0 mm thick was used as a bio-coating for the cornea. On days 3, 7, and 9 from the beginning of the experiment, the results were histologically tested.

**Results:**

On days 3 and 7 of the experiment, histological investigations did not find any structural changes of the anterior segment of the eye and differences between the scaffolds of various thicknesses. The stroma, Descemet’s membrane, endothelium, and general topography of the intercellular matrix were not altered. On day 9, structural changes of the anterior segment were not revealed either in the experiment with the 1.5 mm thick scaffold. Histological investigations of the specimen with the 2.0 mm scaffold showed alterations in the form of epithelium thickening, signs of pseudostratified basal layer, hyperplasia of the wing cell layer with the increased number of its layers, greater number of cellular elements in the anterior stroma layers. No structural changes of the Descemet’s membrane and corneal endothelium were noted.

**Conclusion:**

The suggested hydrogel scaffold-based bio-coating is subject to self-biodegradation without any sequelae to the eye and its adnexa. Increased thickness of the bio-coating results in deceleration of its biodegradation and enhanced activity of proliferative processes in the epithelium and anterior stromal layers, which is indirect evidence of improved regenerative properties of these tissues.

## Introduction

According to the data of the World Health Organization, at least 2.2 billion people suffer from vision disorder and blindness, with corneal pathology occurring in 4.2 million people [1]. In the Russian Federation, corneal diseases make up 5.9% of all causes of low vision and blindness among adult population [2].

The etiology of corneal lesions is manifold and includes a wide spectrum of infectious, inflammatory, dystrophic, neurotrophic, autoimmune diseases, manifestations of primary and secondary limbal insufficiency, traumas and burns. Many superficial corneal defects respond to the standard methods of conservative treatment with a favorable vision outcome. However, the problem of decelerated reepithelialization of the cornea remains one of the vital in modern ophthalmology and may be accompanied by joining a secondary infection, ulceration and perforation of the cornea, its vascularization, formation of stable opacifications, and, as a consequence, considerable sight impairment and blindness [3–5].

In order to stimulate and speed up tissue healing in different fields of regenerative medicine, including ophthalmology, blood derivatives are used in the form of platelet preparations, which stimulate reparative processes and possess a marked anti-inflammatory effect [6–8]. However, to maintain the concentration of biologically active substances on the eye surface and to reach a stable therapeutic effect, frequent instillations of the preparation are required.

Multiple investigations are devoted to the application of amniotic membrane as a biological coating for the cornea. Transplantation of amniotic membrane facilitates epithelialization of corneal defects, since this membrane serves as a basement membrane for the growth of epithelial cells, provides the epithelium with growth factors and cytokines stimulating cell adhesion and proliferation, and decreasing apoptosis of keratocytes [9–12]. However, this material is difficult to collect, requires expensive donor examinations for infections. Besides, it is not available in the Russia as a medical product.

In recent years, various types of bioengineering constructions of scaffolds, structures serving as a substrate and guide for the restoration and regeneration of tissues, have been proposed. These scaffolds possess high biocompatibility, are capable of maintaining high concentration of substances delivered to the injured tissues and, as compared to the amniotic membrane, can be standardized in the process of fabrication [13].

One of the scaffold’s function is creation of the medium imitating natural extracellular matrix (ECM). Hydrogel scaffolds possess self-supporting viscoelastic 3D mesh structure and biological activity, which allows for the generation of adhesive complexes between the cells and ECM. These properties prompted us the idea to evaluate the use of hydrogel scaffolds for treatment of eye diseases associated with long-term non-healing defects of corneal surface. This topic has not actually been presented in the literature, and requires separate investigations to assess the safety of this kind of treatment.

**The aim of the investigation** is to study experimentally the effect of the bio-coating based on the original hydrogel biopolymer scaffold on the intact structures of the anterior segment of the eye and its adnexa and to evaluate its safety.

## Materials and Methods

The experimental study was performed on 6 Chinchilla rabbits (6 eyes) weighing 2.5–3.0 kg. The work was carried out in compliance with the European Convention for the Protection of Vertebrate used for Experimental and Other Specific Purposes (passed in Strasburg, March 18, 1986 and adopted in Strasburg, June 15, 2006).

A bio-coating based on the original hydrogel biopolymer scaffold was the object of the study. The scaffold was developed at Privolzhsky Research Medical University (Nizhny Novgorod, Russia) [14]. A composite based on the human blood plasma cryoprecipitate with PEGylated proteins was used for scaffold formation. A 2% solution of collagen (extracted from cod’s skin [15]), pH 7.2–7.4, was added to the PEGylated cryoprecipitate. The scaffold was formed in the conditions of enzymatic hydrolysis. To polymerize the composite, a thrombin-calcium mixture (80 IU/ml of thrombin in 1% CaCl_2_ solution) was added to it. After the formation, scaffolds were placed on the Petri plates with the physiological solution to prevent hydrogel from drying. Scaffolds were stored at the temperature of 2–8°С until use.

Before the experiment, animals were divided into 2 groups: scaffold 1.5 mm thick was used in group 1 (3 rabbits, 3 eyes), 2.0 mm scaffold (3 rabbits, 3 eyes) was used in group 2. Scaffolds of different thicknesses were chosen to assess the rate of their biodegradation on the intact corneal surface. Ophthalmological status was not changed in all experimental animals before the beginning of the experiment.

**Figure 1.**
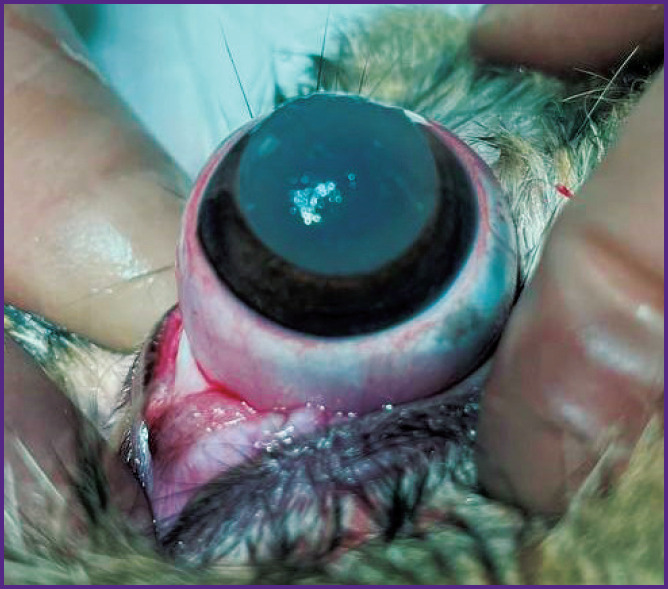
Trepan-cut hydrogel biopolymer scaffold located on the corneal surface

The operative treatment was performed following the rules of asepsis and antiseptics in the condition of drug-induced sleep with subcutaneous injection of 0.1% atropine sulfate solution, intramuscular injection of Zoletil at the dosages proportional to the animal body mass; instillations of Inocain were used locally. After a standard treatment of the surgical field, epibulbar anesthesia and installation of the blepharostat, the third eyelid was removed. A disc of 10.0 mm in diameter was cut from the hydrogel biopolymer scaffold with a trepan and fixed to the cornea of the right eye with four interrupted stitches using Prolene 10-0 suture thread to prevent shifting (Figure 1). The operation was completed by a simple blepharorrhaphia with the Mersilene 5-0. The left eye remained intact.

In the postoperative period, a 0.5% levofloxacin solution was instilled daily 2 times a day to prevent secondary infection. The intensity of the inflammatory reaction of the adnexa and visible structures of the anterior eye segment was clinically evaluated every day.

On days 3, 7, and 9 of the experiment, the specimens were histologically investigated. After the withdrawal of the animals from the experiment and exenteration of the operated eyes, the obtained material was fixed in the 10% neutral buffered formalin, washed in running water, dehydrated with ascending concentration of alcohol and embedded in paraffin. A series of histological sections with hematoxylin and eosin staining was prepared. Samples were studied under the Leica DM LВ2 microscope (Leica, Germany) with 50, 100, 200, 400, and 630× magnification with subsequent photo registration using the slide scanner Leica Aperio CS2 (Leica, USA).

## Results

### Biomicroscopy

After the operation during the entire period of observation, the eyelids remained calm, visible areas of conjunctiva were pale pink, the cornea and underlying structures were not visualized due to the blepharorrhaphia.

On day 3, the scaffold was present in the eyes of all experimental animals on the visible areas of the cornea. On day 7, biodegradation of the scaffolds 1.5 and 2.0 mm thick was visualized and washing out of their fragments from the conjunctival cavity was observed in the form of the white strands during antibiotic instillation. On day 9 of the experiment, the scaffold structures were not seen during examination of the visible structures of the anterior eye segment. This was the reason to withdraw the animals from the experiment on day 9.

Thus, during clinical observation of the animal eyes, negative effect of bio-coating on the structures of the anterior segment of the eye and its adnexa was not noted. No signs of conjunctival cavity infection were observed, purulent discharge was absent.

### Histological investigation

**Figure 2.**
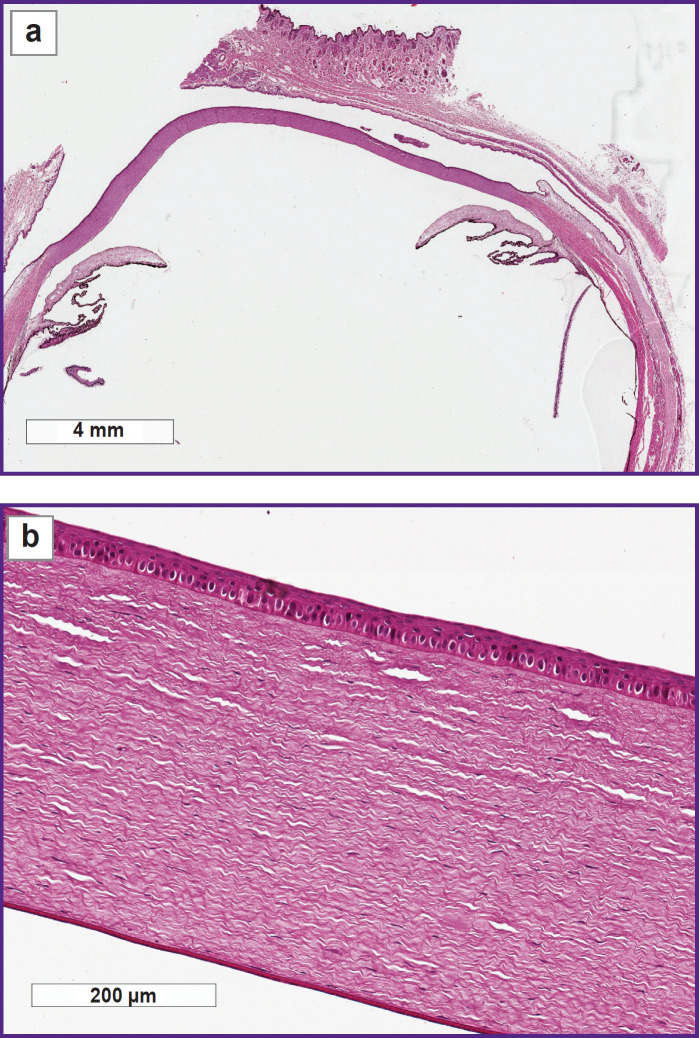
Histological specimen of the rabbit eye on day 3 of the experiment, 1.5 mm scaffold thickness: (a) macrospecimen of the rabbit eye: no changes of the anterior segment are detected; (b) cornea is uniformly epithelialized; stroma is without signs of edema; Descemet’s membrane and endothelium are without specific changes

The histological investigation of the rabbit eye samples on day 3 did not find structural changes of the anterior segment of the eye after applying scaffolds 1.5 and 2.0 mm thick. The cornea was uniformly thick and epithelialized, without changes of the stroma structure, Descemet’s membrane, and endothelium, a general ECM topography was not altered (Figure 2).

**Figure 3.**
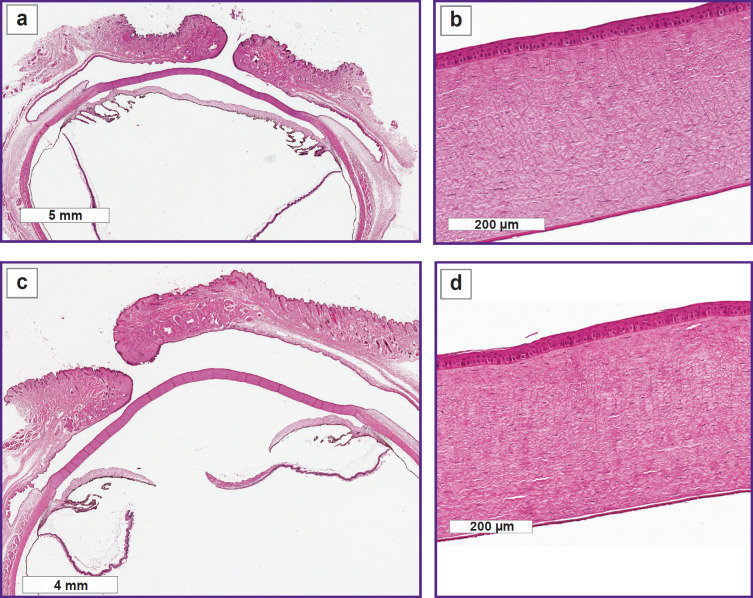
Histological specimen of the rabbit eye on day 7 of the experiment, 1.5 mm (a), (b) and 2.0 mm (c), (d) scaffold thickness: (a), (c) macrospecimen of the rabbit eye: no changes of the anterior segment are detected; (b), (d) cornea is uniformly epithelialized; stroma has no signs of edema; Descemet’s membrane and endothelium are without specific changes

On day 7, the histological investigations also did not detected any structural changes of the anterior segment of the eye in both examined groups (Figure 3).

**Figure 4.**
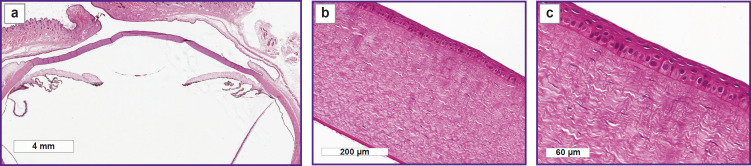
Histological specimen of the rabbit eye on day 9 of the experiment, 1.5 mm scaffold thickness: (a) macrospecimen of the rabbit eye: no changes of the anterior segment are detected; (b), (c) cornea is uniformly epithelialized; stroma has no signs of edema; Descemet’s membrane and endothelium are without specific changes

On day 9 of the experiment, the histological investigation of the rabbit eye specimens with the 1.5 mm thick scaffold did not reveal structural changes of the anterior segment (Figure 4).

**Figure 5.**
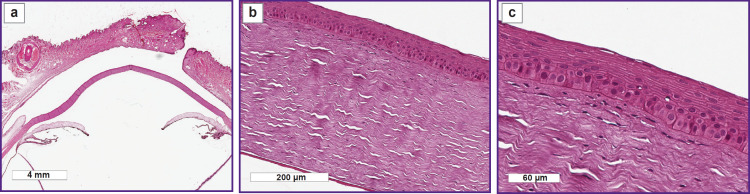
Histological specimen of the rabbit eye on day 9 of the experiment, 2.0 mm scaffold thickness: (a) macrospecimen of the rabbit eye: inclusions on the surface are absent; (b), (c) cornea is uniformly epithelialized; epithelium is thickened due to the greater number of layers; basal layer has the signs of pseudostratification; signs of hyperplasia of the wing cell layer with the number of layers increased up to 10. Stroma is moderately thickened; there is increase in cellular elements in the anterior layers; Descemet’s membrane and endothelium are without specific changes

After the histological processing, traces of the scaffold on the eye surface in the second group on day 9 were not found, however, changes of the anterior epithelium and anterior layers of the corneal stroma were noted. The corneal epithelium was thickened due to the increased number of its layers: the basal layer had the signs of pseudostratification, there were the signs of hyperplasia of the wing cells with the number of the layers increased up to 10. The stroma was moderately thickened with the signs of the increased cellular elements in the anterior layers; Descemet’s membrane and endothelium were without changes (Figure 5).

**Figure 6.**
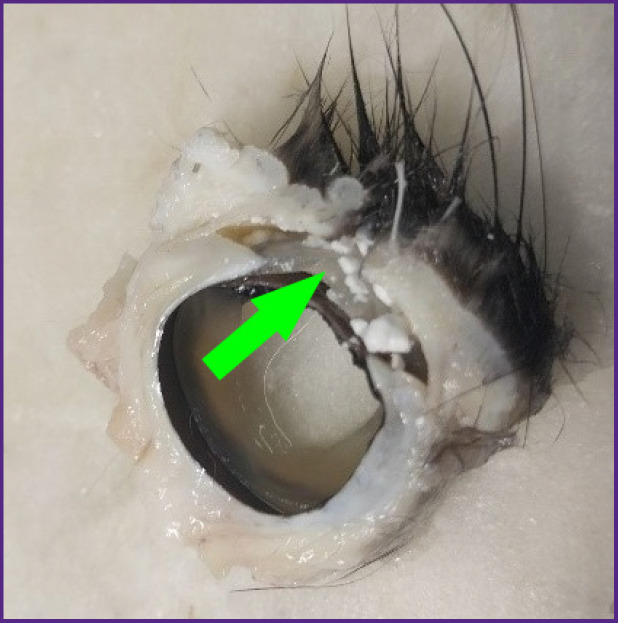
Macrospecimen with residual biopolymer material in the conjunctive cavity (indicated by the arrow)

When cutting out the material with the 2.0 mm thick scaffold, white matt artificial fragments — remnants of biopolymer material subject to biodegradation — were detected on the surface of the cornea and in the conjunctival cavity (Figure 6).

## Discussion

Fibrinogen/fibrin and collagen type I are the main structure-forming proteins of the original hydrogel scaffold [16]. These adhesive peptides may activate integrin receptors on the cell surfaces, bind to the ECM proteins or soluble extracellular ligands.

Collagen type I is a predominant collagen in the majority of tissues in animals of a higher order and is used mostly in medicine. It consists of two α1-chains and α2-chain with a 50 nm fibril diameter [17]. Collagen type 1 makes up 80–90% of keratocyte fibrillar collagen in the corneal structure [18]. Collagens are the predominant component of ECM and basal membranes in all connective tissues including cornea. In addition to this main framework function, collagen can interact with specific receptors on the cell surfaces influencing numerous cell processes including cell adhesion, migration, differentiation, proliferation [19]. Collagens are also involved in capturing, storing, and delivery of growth factors and cytokines, which makes them ideal for creating systems for drug and growth factor transport. Owing to the mentioned receptor interactions and functions of storing and delivery, collagen plays a leading role in wound healing and tissue regeneration [20]. Collagen type I possesses high biocompatibility and ability to biodegradation under the action of endogenous collagenases. Some decay products of exogenous collagen type I–III cause hemotaxis of human fibroblasts. This degradation, in its turn, contributes to the restoration of tissue structure and functions [21].

Blood plasma, which is the base of the presented scaffold composite, is known to contain amino acids necessary for cell growth and proliferation together with some of the key ECM proteins such as fibrinogen and fibronectin [22]. Fibronectin is an adhesive glycoprotein present in lacrimal fluid, basal membrane of the corneal and conjunctival epithelium. It plays an important role in cell migration acting as a temporal matrix along which epithelial cells migrate in the process of epithelial defect regeneration.

Several studies have demonstrated the ability of fibrinogen and fibrin to bind, hold for a long time and slowly release growth factors of various families (PDGF, FGF, TGF-ß) [23, 24]. This physiological feature of the fibrin matrix to act as a depot influences favorably the recovery of the damaged tissues.

In the present study, a long-term adhesion of the corneal epithelium to the scaffold surface contributed to maintaining the viability of the corneal superficial layers (their diminished desquamation) and intensive proliferative activity of the basal epithelium with the increase of wing cell layers. These effects may be largely determined by the signals received by the cells from the biologically active scaffold and maintained by the protein factor complex initially present in the cryoprecipitate of blood plasma, and released by the biopolymer matrix during the period of biodegradation.

The first experiment of using the hydrogel biopolymer has shown that 9 days after fixation of 1.5 mm thick scaffold, its full resorption was noted on the surface of the intact cornea, whereas the residual artificial material remained in the fornixes of the conjunctival cavity in the 2.0 mm thick specimen. The rate of the biomaterial resorption in the wound may depend in many respects on the action of proteolytic enzymes and intensity of the alteration process and inflammation in the injury focus. It should be noted that there was no signs of infection of the ocular surface and adnexa during the entire period of observation of the experimental animals (9 days).

Thus, the original hydrogel scaffold based on the cryoprecipitate of blood plasma and collagen type I does not cause cytotoxic effect on the intact surface of the rabbit cornea.

## Conclusion

The experimental study has shown that the bio-coating based on the original hydrogel biopolymer scaffold is subject to self-biodegradation without consequences for the eye and its adnexa. Increased thickness of the bio-coating led to deceleration of biodegradation and partial preservation of its structures on the eye surface until the 9^th^ day of the experiment. This fact allowed us to demonstrate the activity of proliferative processes in the epithelium and anterior layers of the stroma, which is indirect evidence of improved regenerative properties of the cornea in the suitable microenvironment.
